# Absence of Clinically Meaningful Drug-Drug Interactions with Rezafungin: Outcome of Investigations

**DOI:** 10.1128/spectrum.01339-23

**Published:** 2023-05-08

**Authors:** Shawn Flanagan, Helen Walker, Voon Ong, Taylor Sandison

**Affiliations:** a Cidara Therapeutics, Inc., San Diego, California, USA; b Mundipharma Ltd., Cambridge, United Kingdom; The Ohio State University

**Keywords:** rezafungin, echinocandins, drug-drug interactions, pharmacokinetic profile, cytochrome P450, drug transporters

## Abstract

Rezafungin is a novel once-weekly echinocandin for intravenous injection currently in development for the treatment of *Candida* infections and the prevention of *Candida*, Aspergillus, and Pneumocystis infections in allogeneic blood and marrow transplant recipients. While *in vitro* data indicated that rezafungin exposure was unlikely to be affected by commonly prescribed medicines, interactions resulting in the altered systemic exposure of some drugs coadministered with rezafungin could not be excluded. Two phase 1 open label crossover studies, conducted in healthy subjects, examined drug interactions between rezafungin and multiple drug probe cytochrome P450 (CYP) substrates and/or transporter proteins, immunosuppressants, and cancer therapies. Statistical analysis compared the outcomes for drugs coadministered with rezafungin to those for the drugs administered alone. The geometric mean ratio was reported, and a default 90% confidence interval (CI) no-effect equivalence range of 80 to 125% was used for the maximal plasma concentration (*C*_max_), the area under the curve from time zero to the final sampling time point (AUC_0–_*_t_*), and the AUC from time zero to infinity (AUC_0–∞_). Most probes and concomitant drugs were within the equivalence range. For tacrolimus, ibrutinib, mycophenolic acid, and venetoclax, the AUC or *C*_max_ was reduced (10 to 19%), with lower bounds of the 90% CI values falling outside the no-effect range. The rosuvastatin AUC and *C*_max_ and the repaglinide AUC_0–∞_ were increased (12 to 16%), with the 90% CI being marginally above the upper bound. Overall, the *in vitro* and *in vivo* data demonstrated a low drug interaction potential with rezafungin via CYP substrate/transporter pathways and commonly prescribed comedications, suggesting that coadministration was unlikely to result in clinically significant effects. Treatment-emergent adverse events were typically mild, and rezafungin was generally well tolerated.

**IMPORTANCE** Antifungal agents used to treat life-threatening infections are often associated with severe drug-drug interactions (DDIs) that may limit their usefulness. Rezafungin, a newly approved once-weekly echinocandin, has been shown to be free of DDIs based on extensive nonclinical and clinical testing described in this study.

## INTRODUCTION

Fungal infections represent a significant public health burden, especially in vulnerable patients such as the immunocompromised and critically ill (1). Only three classes of antifungal medications are currently available, and treatment resistance is an ever-present and increasing threat, which has driven the ongoing search for alternative agents to combat invasive infections (2–4). Epidemiological trends in *Candida* species during the past 15 years have revealed a shift toward non-*albicans Candida* species, with the notable rise of the species Candida glabrata and C. auris, presenting diagnostic and therapeutic challenges as well as a greater predisposition to drug resistance (2, 3, 5–12). Estimates concerning drug resistance rates in the United States indicate that C. auris strains show approximately 90% resistance to azoles and around 30% resistance to amphotericin B, while the overall rate of echinocandin resistance remains about 5% (13).

Azoles are widely used to treat invasive candidiasis but are associated with hepatotoxicity, severe skin reactions (e.g., Stevens-Johnson syndrome), and drug-drug interactions (DDIs), making them challenging and complex to use concurrently with various important medicines (including chemotherapies and immunosuppressant drugs) (14–17). Amphotericin B is an intravenous (i.v.) polyene antifungal associated with adverse effects, including nephrotoxicity, infusion reactions and phlebitis, nausea and vomiting, electrolyte disorders, and reversible normocytic anemia (18). Echinocandins are the most recent class of antifungal therapy to have been approved by the Food and Drug Administration (FDA) for the treatment of serious systemic fungal infections and are currently recommended for the first-line treatment of candidemia and invasive candidiasis, partly due to the universal resistance of C. auris strains to azole therapies observed in surveillance programs and studies (13, 19–22). Echinocandins also demonstrate fungistatic activity against Aspergillus (19–21). Currently approved echinocandins are associated with fewer DDIs and toxicities than azoles and amphotericin B, but limitations exist concerning therapeutic exposure with standard dosing (14, 15, 23–30). Some populations (e.g., critically ill, obese, and elderly patients) are subsequently at risk of underdosing in circumstances where *Candida* species with increasing minimum inhibitory concentration (MIC) values are present (particularly C. glabrata and C. auris), and new echinocandin therapies are needed (14, 15, 23–32).

Rezafungin is a novel echinocandin currently in development for the treatment of candidemia and invasive candidiasis as well as for prophylaxis against fungal diseases caused by *Candida*, Aspergillus, and Pneumocystis species in people undergoing allogeneic blood and marrow transplantation (33–35). Rezafungin has demonstrated metabolic and chemical stability, resulting in a low clearance and a long half-life (around 130 h), which allows once-weekly i.v. dosing, compared to daily dosing for the other echinocandins (caspofungin, micafungin, and anidulafungin) (20, 36–42). It has also shown differentiated pharmacodynamic characteristics and exhibits greater tissue distributions than the marketed echinocandins (33, 37–40, 43, 44). The intrinsic properties of rezafungin may enable the necessary penetration for the effective treatment of deep tissue infections, including intra-abdominal and peritoneal candidiasis, which are currently difficult to address with existing echinocandin agents due to lower drug exposures at the approved doses (45–47). While higher doses of marketed echinocandins could provide the therapeutic exposures required to treat deep-seated infections or *Candida* pathogens with elevated MIC values, the accompanying increase in the risk of toxicity may mitigate any therapeutic benefit (24, 28, 38–40, 48–51). However, preclinical data indicate that rezafungin is chemically stable, with no evidence of reactive intermediate formation when incubated in phosphate-buffered saline, and is metabolically stable in liver microsomes and hepatocytes, which may decrease the risk of hepatotoxicity (36).

The echinocandin class has generally been associated with few serious DDIs, and *in vitro* investigations have also indicated minimal DDI potential with rezafungin (4, 36, 52). Here, we report the outcomes from two phase 1, open-label, *in vivo* studies conducted in healthy subjects to examine the effect of rezafungin for injection on the pharmacokinetic (PK) profiles of multiple oral drugs likely to be given concomitantly with rezafungin as well as those commonly used as the substrates for PK interaction studies. In study 1, the DDI potential of rezafungin was assessed clinically using a number of oral probe substrates of cytochrome P450 (CYP) enzymes and/or drug transporter proteins in cases where initial *in vitro* assessments identified (or could not rule out) a potential interaction. Study 2 examined the potential for DDIs in subjects receiving rezafungin for injection alongside oral immunosuppressant and cancer therapies likely to be coadministered with echinocandin/antifungal treatment in clinical practice (cyclosporine, mycophenolate mofetil, venetoclax, and ibrutinib). Studies 1 and 2 compared the PK outcomes for oral probe drugs and concomitant medications following administration alone versus coadministration with rezafungin (with appropriate washout periods allowed between drug administrations to rule out interactions with other agents).

## RESULTS

### Subject characteristics and disposition.

Baseline demographic data for both studies are available in Table S1 in the supplemental material. Study 1 included 26 adults (*n* = 24 male subjects; *n* = 2 female subjects) with a mean age of 39 years (range, 26 to 55 years). All 26 subjects completed the study. Study 2 included 34 subjects with a mean age of 38.6 years (range, 21 to 59 years), and participants were divided into two cohorts: cohort 1 comprised 18 male subjects, and cohort 2 included 16 female subjects (per-protocol population). Overall, four subjects discontinued the study: two cohort 1 subjects discontinued due to an adverse event, and two cohort 2 participants withdrew consent. Therefore, a total of 30 subjects completed study 2.

### Effect of rezafungin on probe substrates for CYP enzymes, transporters, and commonly coadministered drugs.

**FIG 1:**
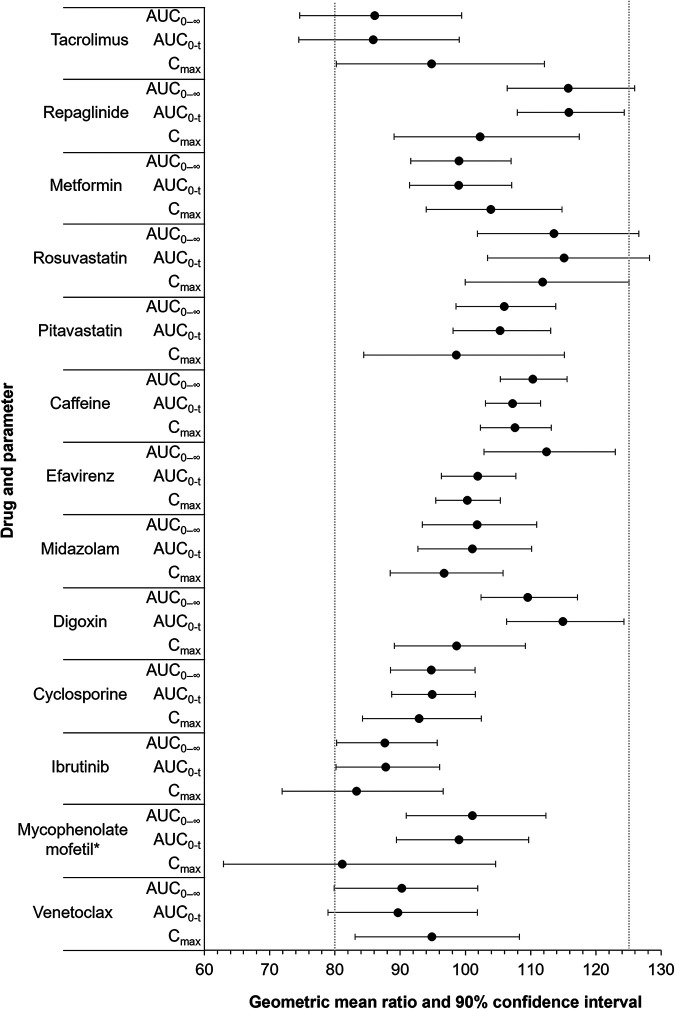
Geometric mean ratios and 90% confidence intervals for drugs coadministered with rezafungin versus the drugs administered alone. Study 1 assessed drug interactions between rezafungin for injection and oral tacrolimus, repaglinide, metformin, rosuvastatin, pitavastatin, caffeine, efavirenz, midazolam, and dioxin. Study 2 examined drug interactions between rezafungin for injection and oral cyclosporine, ibrutinib, mycophenolate mofetil, and venetoclax. * mycophenolic acid was the analyte measured.

**FIG 2:**
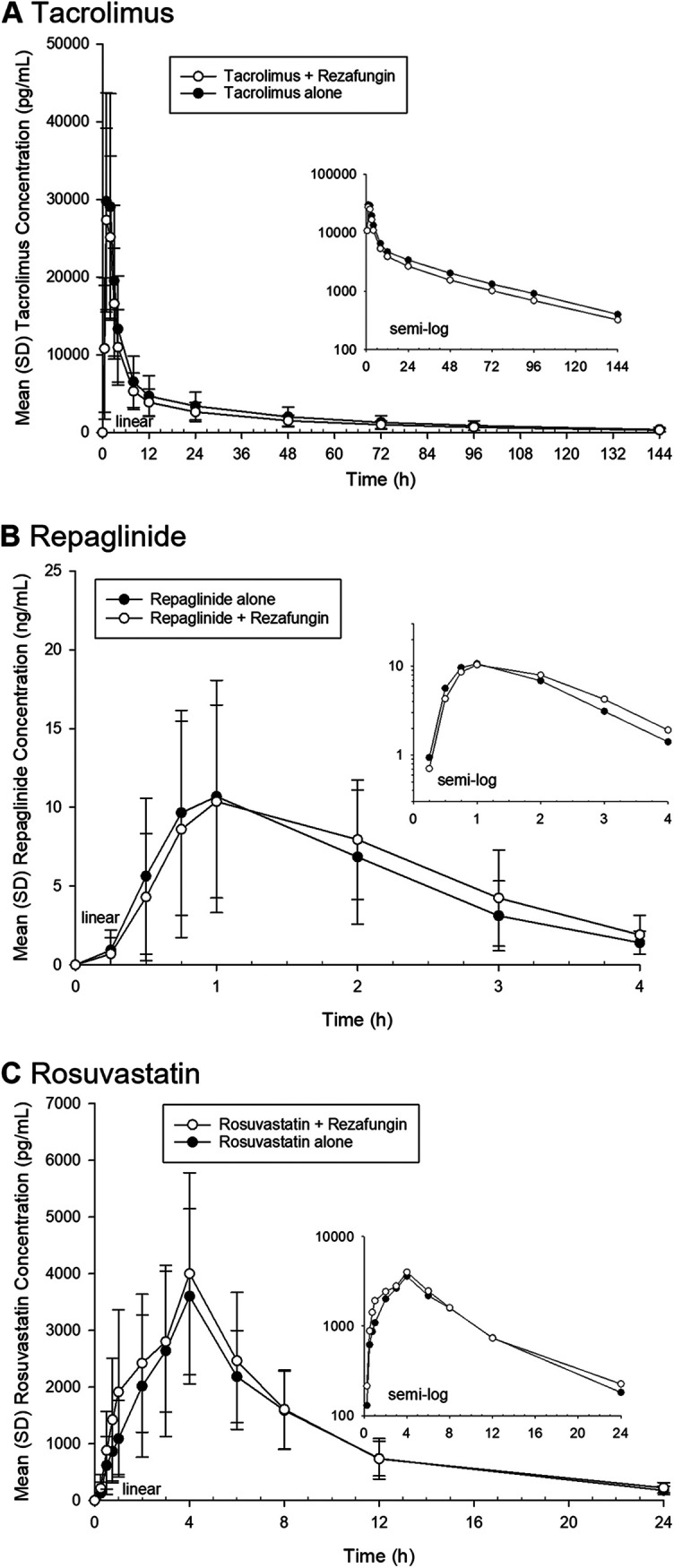
Mean concentration-versus-time profiles for compounds included in study 1 where the 90% confidence intervals for the geometric mean ratios were demonstrated to be outside the no-effect equivalence range of 80 to 125% for the AUC and *C*_max_ parameters. (A) Mean concentration-versus-time profile for tacrolimus coadministered with rezafungin or administered alone. (B) Mean concentration-versus-time profile for repaglinide coadministered with rezafungin or administered alone. (C) Mean concentration-versus-time profile for rosuvastatin coadministered with rezafungin or administered alone.

**FIG 3:**
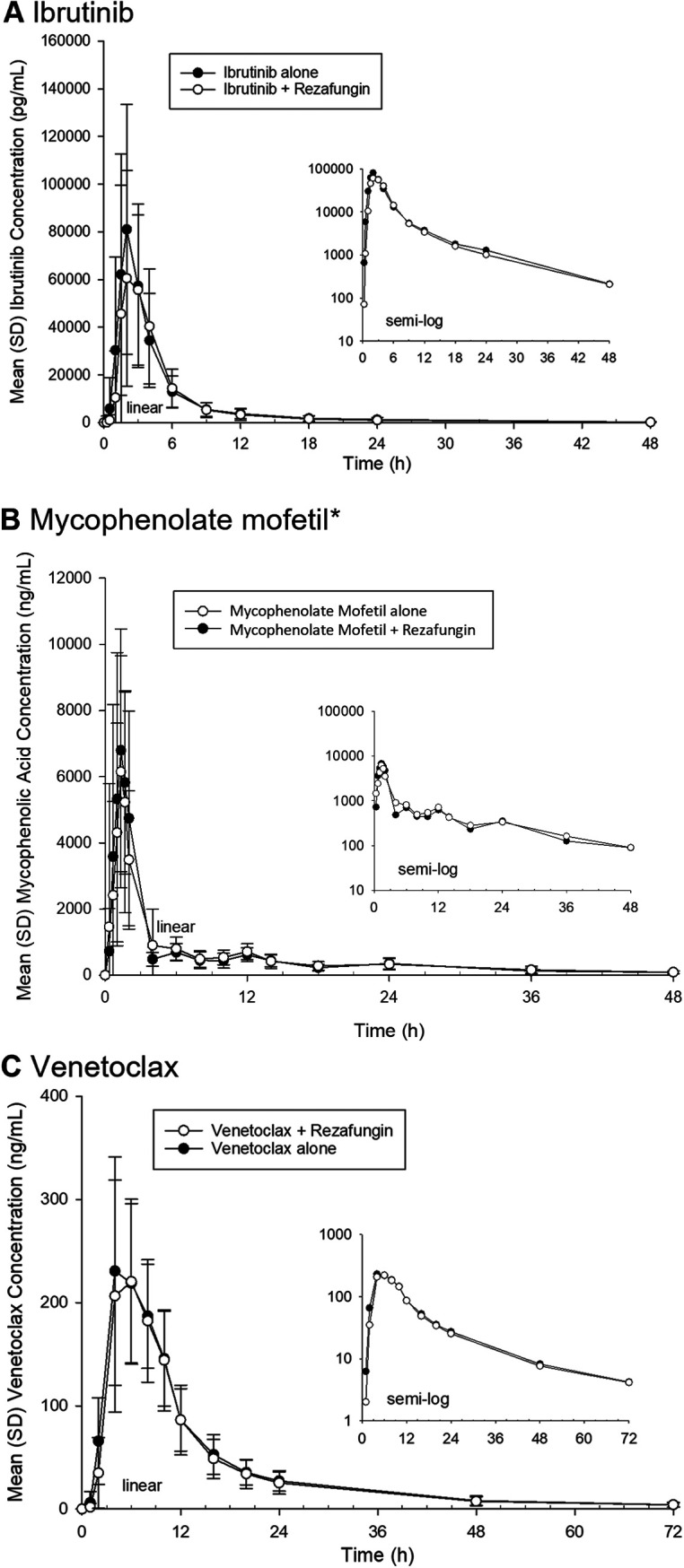
Mean concentration-versus-time profiles for compounds included in study 2 where the 90% confidence intervals for the geometric mean ratios were demonstrated to be outside the no-effect equivalence range of 80 to 125% for the AUC and *C*_max_ parameters. (A) Mean concentration-versus-time profile for ibrutinib coadministered with rezafungin or administered alone. (B) Mean concentration-versus-time profile for mycophenolate mofetil coadministered with rezafungin or administered alone. (C) Mean concentration-versus-time profile for venetoclax coadministered with rezafungin or administered alone. * mycophenolic acid was the analyte measured.

Table 1 provides a combined summary of statistical comparisons for PK parameters related to assessments conducted in studies 1 and 2, and Fig. 1 presents a forest plot of the geometric mean ratios (GMRs) and 90% confidence intervals (CIs) for the maximal plasma concentration (*C*_max_) and the area under the curve (AUC). Fig. 2 and 3 present the mean concentration-time profiles with or without rezafungin coadministration for compounds whose confidence intervals exceeded the default no-effect boundary in study 1 and study 2, respectively. Table 2 provides a summary of PK outcomes for each of the drugs tested in study 1 and study 2 and the potential dosing implications for each of these agents when coadministered with rezafungin in clinical practice.

**TABLE 1:** Summary of statistical comparisons of study 1 and 2 results regarding plasma PK parameters following the coadministration of the probe/test drugs with rezafungin versus the administration of test drugs alone^*a*^

Study, treatment comparison, and parameter	Geometric LSM value		Geometric mean ratio (%)	90% CI of the geometric mean ratio (%)
	+ rezafungin	Alone		
Study 1				
Tacrolimus + rezafungin at 600 mg/tacrolimus alone				
*C*_max_ (pg/mL)	28,909.40	30,489.62	94.82	80.20–112.09
AUC_0–_*_t_* (pg · h/mL)	266,002.13	309,698.20	85.89	74.47–99.07
AUC_0–∞_ (pg · h/mL)	283,328.47	328,990.49	86.12	74.59–99.43
Repaglinide + rezafungin at 600 mg/repaglinide alone				
*C*_max_ (ng/mL)	12.13	11.86	102.26	89.05–117.43
AUC_0–_*_t_* (ng · h/mL)	23.47	20.25	115.87	107.96–124.35
AUC_0–∞_ (ng · h/mL)	25.16	21.74	115.75	106.39–125.93
Metformin + rezafungin at 400 mg/metformin alone				
*C*_max_ (ng/mL)	1,115.98	1,074.27	103.88	94.00–114.81
AUC_0–_*_t_* (ng · h/mL)	7,257.55	7,333.63	98.96	91.46–107.08
AUC_0–∞_ (ng · h/mL)	7,373.31	7,446.67	99.02	91.64–106.98
Rosuvastatin + rezafungin at 400 mg/rosuvastatin alone				
*C*_max_ (pg/mL)	3,683.66	3,294.35	111.82	99.99–125.05
AUC_0–_*_t_* (pg · h/mL)	31,027.38	26,949.43	115.13	103.39–128.20
AUC_0–∞_ (pg · h/mL)	32,386.11	28,521.34	113.55	101.85–126.59
Pitavastatin + rezafungin at 400 mg/pitavastatin alone				
*C*_max_ (ng/mL)	35.72	36.23	98.59	84.40–115.17
AUC_0–_*_t_* (ng · h/mL)	81.54	77.44	105.30	98.08–113.05
AUC_0–∞_ (ng · h/mL)	90.87	85.78	105.93	98.56–113.86
Caffeine + rezafungin at 400 mg/caffeine alone				
*C*_max_ (ng/mL)	2,232.88	2,075.44	107.59	102.31–113.14
AUC_0–_*_t_* (ng · h/mL)	18,475.36	17,230.22	107.23	103.09–111.53
AUC_0–∞_ (ng · h/mL)	20,853.11	18,897.70	110.35	105.34–115.59
Efavirenz + rezafungin at 400 mg/efavirenz alone				
*C*_max_ (ng/mL)	300.17	299.30	100.29	95.46–105.37
AUC_0–_*_t_* (ng · h/mL)	7,698.46	7,557.26	101.87	96.32–107.74
AUC_0–∞_ (ng · h/mL)	16,156.14	14,368.15	112.44	102.83–122.96
Midazolam + rezafungin at 400 mg/midazolam alone				
*C*_max_ (ng/mL)	8.70	9.00	96.73	88.48–105.75
AUC_0–_*_t_* (ng · h/mL)	21.62	21.39	101.06	92.73–110.13
AUC_0–∞_ (ng · h/mL)	23.67	23.25	101.78	93.39–110.92
Digoxin + rezafungin at 400 mg/digoxin alone				
*C*_max_ (ng/mL)	0.84	0.85	98.64	89.10–109.20
AUC_0–_*_t_* (ng · h/mL)	13.96	12.14	114.95	106.33–124.27
AUC_0–∞_ (ng · h/mL)	18.94	17.29	109.55	102.41–117.18
Study 2				
Cyclosporine + rezafungin at 400 mg/cyclosporine alone				
*C*_max_ (ng/mL)	919.42	989.81	92.89	84.23–102.44
AUC_0–_*_t_* (ng · h/mL)	4,537.99	4,781.06	94.92	88.72–101.54
AUC_0–∞_ (ng · h/mL)	4,740.20	5,001.93	94.77	88.50–101.47
Ibrutinib + rezafungin at 200 mg/ibrutinib alone				
*C*_max_ (ng/mL)	65,547.61	78,650.01	83.34	71.92–96.58
AUC_0–_*_t_* (ng · h/mL)	240,725.76	274,272.45	87.77	80.19–96.07
AUC_0–∞_ (ng · h/mL)	243,135.30	277,456.39	87.63	80.25–95.69
Mycophenolate mofetil + rezafungin at 200 mg/mycophenolate mofetil alone^*b*^				
*C*_max_ (ng/mL)	7,740.58	9,541.20	81.13	62.91–104.63
AUC_0–_*_t_* (ng · h/mL)	25,054.90	25,299.70	99.03	89.42–109.68
AUC_0–∞_ (ng · h/mL)	28,257.00	27,954.20	101.08	90.96–112.33
Venetoclax + rezafungin at 200 mg/venetoclax alone				
*C*_max_ (pg/mL)	240.44	253.51	94.84	83.08–108.26
AUC_0–_*_t_* (pg · h/mL)	2,693.83	3,004.70	89.65	78.92–101.85
AUC_0–∞_ (pg · h/mL)	2,781.57	3,082.58	90.24	79.91–101.89

a Abbreviations: AUC_0–∞_, area under the curve from time zero to infinity; AUC_0–_*_t_*, area under the curve from time zero to the final sampling time point; CI, confidence interval; *C*_max_, maximum plasma concentration. The geometric least-squares mean (LSM) ratio (percent) was calculated as (geometric LSM of the drug + rezafungin)/(geometric LSM of the drug administered alone) × 100.

b Mycophenolic acid was the analyte measured.

**TABLE 2:** Summary of the pharmacokinetic outcomes for each of the probe/test drugs coadministered with rezafungin, compared with administration alone, and potential dosing implications^*d*^

Drug	Possible mechanism(s)	Observation^*a*^		Suggested action
		*C*_max_	AUC	
Tacrolimus	CYP3A4, P-gp	↔	↓ ~14%	No change in dose
Repaglinide	CYP2C8, OATP	↔	↑ ~16% (0–∞)	No change in dose
Metformin	OCT, MATEs	↔	↔	No change in dose
Rosuvastatin	BCRP, OATP	↑ ~12%	↑ ~15%	No change in dose
Pitavastatin	OATP	↔	↔	No change in dose
Caffeine	CYP1A2	↔	↔	No change in dose
Efavirenz	CYP2B6	↔	↔	No change in dose
Midazolam	CYP3A4	↔	↔	No change in dose
Digoxin	P-gp	↔	↔	No change in dose
Cyclosporine	CYP3A4, P-gp	↔	↔	No change in dose
Ibrutinib	CYP3A4, P-gp, BCRP	↓ ~17%	↔	No change in dose
Mycophenolate mofetil^*c*^	Other^*b*^	↓ ~19%	↔	No change in dose
Venetoclax	CYP3A4, P-gp	↔	↓ ~10%	No change in dose

a The magnitude of the change indicates the ratio of the geometric mean PK parameter for the test (with rezafungin) relative to the reference (drug alone).

b Drugs affecting absorption or enterohepatic recirculation.

c Mycophenolic acid was the analyte measured.

d Abbreviations: AUC, area under the concentration-time curve (refers to both the AUC from time zero to the time point of the last quantifiable sample and the AUC extrapolated to time infinity, unless otherwise noted); *C*_max_, maximum concentration; ↔, no change (the ratio of the PK parameter value varied by up to ~10%, and the 90% CI was within 80 to 125%); ↓, decrease in exposure; ↑, increase in exposure.

Study 1 results showed that the coadministration of i.v. rezafungin with the oral drugs efavirenz (probe substrate for CYP2B6), midazolam (CYP3A4 substrate), caffeine (CYP1A2 substrate), metformin (substrate for organic cation transporter 1 [OCT-1], OCT-2, multidrug and toxin extrusion 1 [MATE-1], and MATE-2), pitavastatin (organic anion-transporting polypeptide [OATP] substrate), and digoxin (P-glycoprotein [P-gp] substrate) did not result in statistically significant changes in the exposure of the probe drugs compared with the dosing of each drug alone. Statistical analysis demonstrated that the 90% CIs of the GMRs related to the *C*_max_, the AUC from time zero to the final sampling time point (AUC_0–_*_t_*), and the AUC from time zero to infinity (AUC_0–∞_) were all within the default no-effect equivalence range of 80 to 125% for these drugs.

The 90% CIs of the GMRs for the *C*_max_ of oral tacrolimus (CYP3A4 and P-gp substrates) and the *C*_max_ and AUC_0–_*_t_* of oral repaglinide (CYP2C8 substrate and OATP transporter substrate) following coadministration with rezafungin were within the no-effect boundary, compared with administration alone. However, the AUC_0–_*_t_* and AUC_0–∞_ for tacrolimus were reduced by approximately 14%, and the lower bound of the 90% CI of the GMR for each parameter fell below the lower boundary for no effect (AUC_0–_*_t_*, 74.47%; AUC_0–∞_, 74.59%). The AUC_0–∞_ of repaglinide was increased by approximately 16% with rezafungin coadministration, compared with administration alone, and the upper bound of the 90% CI of the GMR was above the no-effect limit (125.93%). The administration of oral rosuvastatin (breast cancer resistance protein [BCRP] and OATP substrate) with rezafungin resulted in increases in the *C*_max_, AUC_0–_*_t_*, and AUC_0–∞_ of approximately 12%, 15%, and 14%, respectively, compared with administration alone, and the upper bound of the 90% CIs of the GMRs for these parameters fell outside the upper no-effect limit (*C*_max_, 125.05%; AUC_0–_*_t_*, 128.20%; AUC_0–∞_, 126.59%).

Study 2 outcomes revealed that the coadministration of i.v. rezafungin did not result in a statistically significant change in the exposure of oral cyclosporine (*C*_max_, AUC_0–_*_t_*, and AUC_0–∞_) compared with administration alone. When rezafungin was coadministered with oral ibrutinib or oral mycophenolate mofetil, the GMRs and 90% CIs for the AUC parameters fell within the no-effect boundary compared to the dosing of these drugs alone. However, the *C*_max_ values of ibrutinib and mycophenolic acid were reduced by approximately 17% and 19%, respectively, with rezafungin coadministration, and the lower bound of the 90% CI of the GMR fell below the no-effect boundary in each case (ibrutinib, 71.92%; mycophenolic acid, 62.91%). The data showed that the *C*_max_ of oral venetoclax was unaffected by i.v. rezafungin coadministration, although the AUC_0–_*_t_* and AUC_0–∞_ were reduced by approximately 10%, and the lower bounds of the 90% CIs of the GMRs fell just outside the no-effect boundary (AUC_0–_*_t_*, 78.92%; AUC_0–∞_, 79.91%).

### Safety.

The majority of clinical laboratory results, vital signs, and 12-lead electrocardiograms (EKGs) remained within normal parameters throughout both studies. The study 1 safety population reported 41 treatment-emergent adverse events (TEAEs) in 14 subjects, which were mild (*n* = 39; 95.1%) or moderate (*n* = 2; 4.9%) in intensity. The rezafungin safety population reported 33 TEAEs in 13 subjects, most of which (*n* = 31; 93.9%) were mild and 2 (6.1%) of which were moderate. All TEAEs were resolved without the discontinuation of rezafungin, and the majority resolved spontaneously or required nonpharmacological intervention. The most common TEAEs included mild diarrhea, abdominal pain, constipation, feeling hot, somnolence, and headache (Table S2). There were no serious TEAEs or deaths or discontinuations due to a TEAE related to study 1.

Overall, 10 subjects (29.4%) included in study 2 experienced TEAEs following the administration of drugs without rezafungin, and 15 subjects (46.9%) experienced TEAEs following rezafungin coadministration. The most commonly reported TEAEs were headache, feeling hot, and nausea (Table S3). Most TEAEs (*n* = 20 [90.9%] without rezafungin; *n* = 35 [87.5%] with rezafungin) were of a mild intensity. Two subjects each reported a severe TEAE, abdominal pain (deemed by the investigator to be related to rezafungin and venetoclax) and esophagitis (deemed by the investigator to be related to cyclosporine), which occurred prior to the first dose of rezafungin. One subject withdrew due to esophagitis, and another subject left the study following an infusion reaction. There were no deaths or serious adverse events during the study period.

## DISCUSSION

The results from the two phase 1 open-label crossover studies in healthy subjects reported here (study 1 and study 2) provided key insights regarding the likelihood of DDIs between rezafungin for injection, a novel echinocandin, and CYP substrates and/or transporter proteins, immunosuppressants, and cancer therapies. Once-weekly i.v. infusions of rezafungin, given either as a 600-mg loading dose followed by two 400-mg maintenance doses or as a 400-mg loading dose followed by two 200-mg maintenance doses, were generally well tolerated, with typically mild TEAEs that resolved during the study period. These results reflect published preclinical data and clinical safety outcomes for rezafungin and provide reassurance regarding its coadministration with commonly used medications (34, 36, 37, 41, 42).

Initial *in vitro* assessments indicated that rezafungin was unlikely to be a victim of clinically meaningful DDIs given that it was found to be metabolically stable in incubations with hepatocytes and microsomes and was not observed to be a substrate of any of the major drug transporter proteins tested. The rezafungin PK results from these studies with one or more concomitant medications were consistent with the PK results from other studies when rezafungin was administered alone (Flanagan, Ong, Sandison, unpublished data). Initial *in vitro* assessments could not rule out the risk of rezafungin affecting the exposures of concomitantly administered drugs, and additional investigations were therefore required to examine potential clinically meaningful drug interactions. *In vivo* DDI assessments conducted during study 1 were designed using a three-drug cocktail approach and the insights gained through the initial *in vitro* studies. This approach allowed the simultaneous administration of multiple drugs that are known substrates of CYP enzymes and/or transporter proteins. The cocktails were administered separately, with and without rezafungin, to ensure the washout of the drugs in the cocktail between administrations. For six of the nine substrate drugs (metformin, pitavastatin, caffeine, efavirenz, midazolam, and digoxin), there was an absence of DDIs as defined by the GMRs and 90% CIs of the exposure parameters (*C*_max_ and AUC) falling within the default no-effect boundary of 80 to 125% for each drug administered with rezafungin compared to administration alone.

Rezafungin produced small decreases (approximately 14%) in the AUC_0–_*_t_* and AUC_0–∞_ of tacrolimus (CYP3A4 and P-gp substrate), which was administered in the first cocktail, and an interaction could not be ruled out because the lower limit of the 90% CI of the GMR fell below the lower no-effect boundary for each AUC parameter. However, the direction of change indicated that no inhibition of CYP3A4 or P-gp was observed. In addition, rezafungin did not significantly alter the PK of midazolam (a CYP3A4-sensitive index substrate) and digoxin (a P-gp substrate drug). As these probes were administered in the third cocktail of study 1 (day 15) when the subjects had previously received 600 mg rezafungin on day 1 and 400 mg on day 10 (doses that are higher than the proposed clinical dosing regimen of 400 mg on day 1 and 200 mg weekly thereafter), the data suggest that the decrease in the overall exposure of tacrolimus observed following the administration of rezafungin in cocktail 1 was not a result of altered CYP3A4-mediated metabolism or P-gp-mediated transport due to induction. The small decrease in tacrolimus exposure was not clinically meaningful. When repaglinide (a CYP2C8-sensitive index substrate and OATP substrate) was coadministered with rezafungin, the GMR for the AUC_0–∞_ was increased for repaglinide by approximately 16%, and the upper bound of the 90% CI was just above the upper limit of the no-effect boundary (125.93%), compared to administration alone. The coadministration of rosuvastatin (BCRP and OATP substrate) with rezafungin resulted in increases in the *C*_max_, AUC_0–_*_t_*, and AUC_0–∞_ by approximately 12%, 15%, and 14%, respectively, and the upper limit of the 90% CI of the GMR was just above the no-effect boundary in each case. While OATP is involved in the disposition of both repaglinide and rosuvastatin, pitavastatin (also an OATP probe substrate) exposure was not affected by coadministration with rezafungin, indicating that the inhibition of OATP is unlikely to account for the increase in the exposures of repaglinide and rosuvastatin. Furthermore, these changes in exposures were not deemed clinically meaningful.

Study 2 was designed to assess whether rezafungin was associated with any clinically meaningful interactions when coadministered with immunosuppressant and oncology drugs likely to be given alongside echinocandin/antifungal treatments in clinical practice, namely, cyclosporine, mycophenolate mofetil, ibrutinib, and venetoclax. This study confirmed that no unexpected interactions were observed with rezafungin coadministration, as indicated by the absence of relevant mechanistic interactions shown in study 1. Rezafungin did not affect cyclosporine exposure. There was no effect on the AUCs of ibrutinib and mycophenolic acid when coadministered with rezafungin, but the *C*_max_ values of ibrutinib and mycophenolic acid were reduced by 17% and 19%, respectively, when ibrutinib and mycophenolate mofetil were administered with rezafungin, with the lower limit of the 90% CI of the GMR falling below the no-effect range. Given the lack of an effect on the AUC and the magnitude of the effect on the *C*_max_, it was considered unlikely that any clinically meaningful interaction would occur upon the coadministration of rezafungin and ibrutinib or mycophenolate mofetil. The administration of venetoclax with rezafungin did not result in an effect on the *C*_max_ of venetoclax, but the AUC parameters were reduced (approximately 10%), and the lower bounds of the 90% CIs of the GMRs were only just outside the no-effect boundary of 80%. The size of the effect on venetoclax AUC parameters and the lack of an effect on the *C*_max_ indicated a low potential for any clinically meaningful interaction between rezafungin and venetoclax.

In summary, rezafungin had no or minimal effects on the exposure of probe drugs for the following CYP substrates/transporter proteins: CYP2B6 (efavirenz); CYP3A4 (midazolam and tacrolimus); CYP1A2 (caffeine); CYP2C8 (repaglinide); P-gp (digoxin and tacrolimus); OCT-1, OCT-2, MATE-1, and MATE-2 (metformin); OATP (pitavastatin, rosuvastatin, and repaglinide); and BCRP (rosuvastatin). No clinically meaningful interactions were identified upon the coadministration of rezafungin with tacrolimus, cyclosporine, ibrutinib, mycophenolate mofetil, and venetoclax. These results demonstrate the low potential for CYP substrate-mediated and drug transporter-mediated DDIs with rezafungin and also reveal the minimal impact on PK parameters with commonly coprescribed medications. These findings also provide evidence to support the safety-and-tolerability profile of rezafungin when coadministered with commonly used drugs and indicate that the prescribing of rezafungin should not require dose adjustments for concomitant medications. Rezafungin has an advantage over caspofungin with regard to DDI liability, as caspofungin has warnings in its U.S. prescribing information based on the outcomes of two clinical studies demonstrating that cyclosporine increased the AUC of caspofungin by approximately 35%, resulting in the elevation of liver enzymes, and rifampicin reduced the trough concentrations of caspofungin by 30% (53). There is also a warning that other hepatic CYP enzyme inducers may result in clinically meaningful reductions in caspofungin concentrations, although the inducible drug clearance mechanism that is involved in caspofungin disposition is unknown (53). The lack of drug interactions observed with rezafungin is in stark contrast to the numerous DDI challenges widely associated with azole antifungals due to their effects as the substrates for and inhibitors of CYP enzymes and inhibitors of membrane transporter proteins (16, 17). By exhibiting a lack of clinically meaningful DDIs, rezafungin offers a significant advantage over azole antifungals, especially in immunocompromised patients receiving medications with a narrow therapeutic index that may interact with azole antifungals (16, 17).

## MATERIALS AND METHODS

### Initial *in vitro* studies.

Initial *in vitro* studies demonstrated that rezafungin produced weak inhibition of the CYP enzymes CYP2C8 and CYP3A4 (50% inhibitory concentration [IC_50_] of >25 μM) in human liver microsomal preparations (see Table S4 in the supplemental material). Rezafungin moderately inhibited (IC_50_ of >6 μM) several uptake transporters, especially OCT-1, MATE-1, and OATP-1B3, and inhibition of P-gp and BCRP could not be ruled out due to the sensitivity of the cells to rezafungin. Induction studies could not be conducted *in vitro* at concentrations high enough to rule out risks due to solubility limits. Therefore, potential clinical interactions were investigated further *in vivo* in study 1.

### Study design.

**FIG 4:**
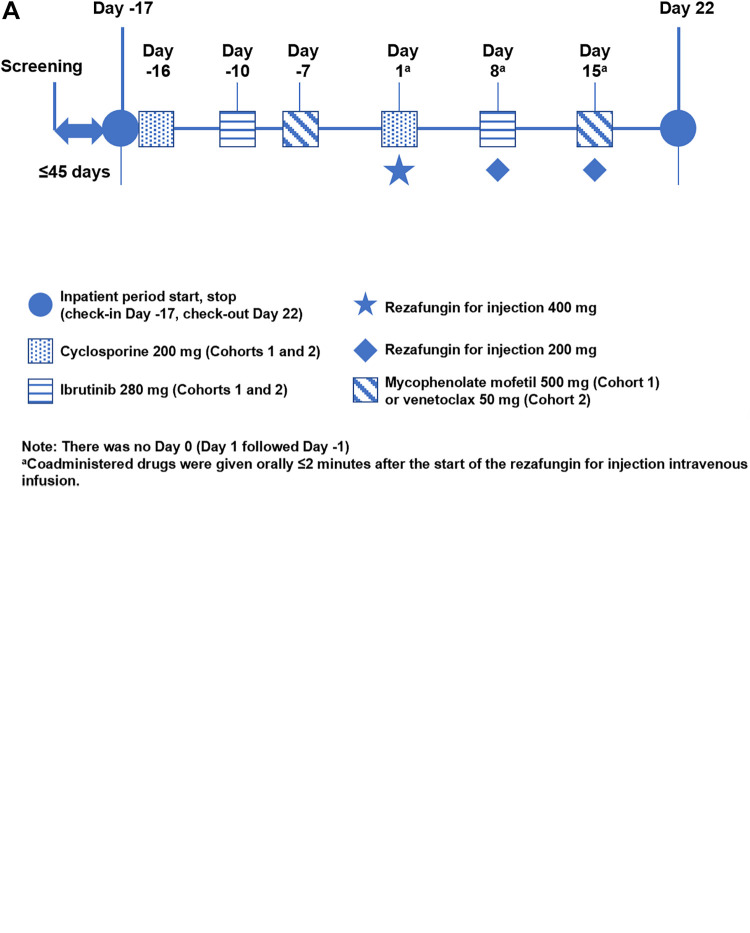
Study designs for the two reported phase 1, single-center, open-label, crossover inpatient studies conducted in healthy subjects. (A) Study 1 assessed drug interactions between rezafungin for injection and oral drugs commonly used as the probe substrates for assessing PK interactions. Probe/test drugs were administered as a cocktail containing two or more drugs once before receiving rezafungin and once after receiving rezafungin on a schedule designed to allow washout between doses and limit interactions with other drugs. (B) Study 2 examined drug interactions between rezafungin for injection and oral immunosuppressant or oncology drugs commonly coadministered with echinocandin/antifungal therapy. Drugs tested for interactions with rezafungin were each administered twice (on a schedule designed to allow washout between doses): once alone and once while receiving rezafungin for injection.

Figure 4 shows the design and schedule for study 1 and study 2. Both were phase 1, single-center, open-label, crossover inpatient studies that were conducted in the United States in healthy adults (study 1, aged 26 to 55 years; study 2, aged 21 to 59 years). The studies were compliant with the International Council for Harmonisation of Technical Requirements for Pharmaceuticals for Human Use E6 guideline for good clinical practice (54) and the Declaration of Helsinki. The study protocols were approved by the institutional review board associated with the study site, and all subjects provided written informed consent.

The primary objective of study 1 was to assess the effect of rezafungin on the PK profiles of multiple drugs known to be substrates of CYP enzymes and/or transporters. The secondary objective was to assess the safety and tolerability of rezafungin. The primary objective of study 2 was to assess the effect of rezafungin on the PK profiles of four drugs likely to be coadministered with echinocandins in clinical practice: cyclosporine, ibrutinib, mycophenolate mofetil, and venetoclax. The secondary objectives were to assess the safety and tolerability of rezafungin and to characterize the PK of rezafungin.

### Rezafungin dosing in study 1 and study 2.

The rezafungin doses used during the study were intended to simulate clinically relevant dosing regimens. Study 1 was conducted earlier in the clinical program and used a loading dose of 600 mg and a maintenance dose of 400 mg, which would have covered the 400-mg once-weekly dosing regimen that could have been selected for the phase 3 clinical efficacy studies. Study 2 was conducted later in the clinical program and used a loading dose of 400 mg and a maintenance dose of 200 mg, which could have been selected as the proposed commercial dosing regimen. In each study, the higher loading dose was designed to approximate a steady-state blood concentration reflecting the maintenance dose and enable DDI testing with the first dose.

### Study 1 procedures.

After screening, subjects were admitted to the clinical research unit (CRU) prior to the first dose of rezafungin (from day −22) and were discharged from the CRU on day 22. Nine oral substrate drugs were administered as part of a cocktail containing two or more drugs once before and once after receiving rezafungin on a schedule designed to allow the washout of the drugs in the cocktail between doses and limit interactions with other drugs (Fig. 4A). The substrate drugs and the associated CYP enzymes and/or transporters tested comprised 1 mg repaglinide (CYP2C8 and OATP), 500 mg metformin (OCT-1, OCT-2, MATE-1, and MATE-2), 5 mg rosuvastatin (BCRP and OATP), 2 mg pitavastatin (OATP), 100 mg caffeine (CYP1A2), 50 mg efavirenz (CYP2B6), 2 mg midazolam (CYP3A4), 0.25 mg digoxin (P-gp), and 5 mg tacrolimus (CYP3A4 and P-gp). A loading dose of rezafungin for injection (600 mg) was administered over 1 h on day 1, followed by two once-weekly 400-mg doses over 1 h on days 10 and 15. Due to a short delay in the availability of the investigation study drug, the second once-weekly dose of rezafungin and the second set of oral substrate drugs were administered on day 10 instead of day 8, although the time between doses remained sufficient for all originally planned PK samplings through 96 h postdose and for the adequate washout of the substrate drugs. The total duration of treatment was up to 89 days, including 45 days of screening and 44 days of treatment in the CRU.

### Study 2 procedures.

Male (cohort 1) and female (cohort 2) participants were dosed with oral drugs (200 mg cyclosporine, 280 mg ibrutinib, 500 mg mycophenolate mofetil [cohort 1 only], and 50 mg venetoclax [cohort 2 only]) alone and in combination with rezafungin for injection in a sequential crossover design. After screening, subjects were admitted to the CRU on day −17 (prior to the first rezafungin dose) and were discharged on day 22. Rezafungin was dosed via i.v. infusion over 1 h at 400 mg on day 1 and 200 mg on day 8 and day 15. The oral drugs to be tested for interactions were each administered twice on a schedule designed to allow washout between doses: once alone and once while receiving rezafungin (Fig. 4B). The total duration of treatment was approximately 84 days, which included 45 days of screening and 39 days of treatment in the CRU.

### Bioanalytical and PK assessments.

In study 1, predose PK blood samples were collected on each dosing day ≤60 min prior to the administration of the substrate drugs with or without rezafungin. Multiple blood samples were collected relative to each dose, with collection times ranging from 15 min to 168 h postdose, depending on the PK profile of each probe substrate drug (Table S5). For study 2, multiple blood samples were collected, and the timing of collection varied according to the PK profile of each coadministered drug from predose to 96 h postdose (Table S6). PK blood samples were collected for rezafungin at sample times ranging from predose to 168 h postdose.

For both studies, validated liquid chromatography-tandem mass spectrometry (LC-MS/MS) methods were used for PK assessments to analyze the concentrations of rezafungin and coadministered drugs. All plasma concentrations and actual blood sampling times were subjected to noncompartmental PK analysis. PK parameter calculations were performed using noncompartmental analysis (NCA) (Phoenix WinNonlin version 8.0 or higher). The PK parameters analyzed included *C*_max_; the time to reach the maximum observed plasma concentration (*T*_max_); AUC_0–_*_t_* (using the log-linear trapezoidal rule, where *t* was the last time point with a measurable concentration); AUC_0–∞_ (calculated as AUC_0–_*_t_* + *C*_last_/λ*_z_*); the AUC from 0 to 168 h (AUC_0–168_); the percentage of AUC_0–∞_ extrapolated (AUC_%Extrap_); the terminal elimination rate constant, determined by linear regression of the terminal linear segment of the log concentration-versus-time curve (λ*_z_*); and the elimination half-life (*t*_1/2_), calculated as ln(2)/λ*_z_*.

### Safety assessments.

In both studies, the safety population included all subjects who received any amount of the study drug. In study 1, the rezafungin safety population included all subjects who received any amount of rezafungin. Safety assessments included vital signs (temperature, heart rate, blood pressure, and respiratory rate), safety laboratory evaluations (hematology assessment, serum chemistry panel, and urinalysis), physical examinations (complete and symptom directed), and EKGs.

### Statistical analysis.

The primary analysis of the drug interaction effect on substrate drugs following rezafungin administration was assessed by the ratio of the geometric least-squares (LS) mean of each substrate drug administered with rezafungin to that without rezafungin. The corresponding 90% CIs were calculated for the natural log-transformed parameters *C*_max_, AUC_0–_*_t_*, and AUC_0–∞_. PK parameters were statistically analyzed using an analysis of variance (ANOVA) model. The results were described relative to the default predefined no-effect boundary range (equivalence range) of 80% to 125%. No DDI was typically demonstrated if the 90% CIs for systemic exposure ratios fell completely within these no-effect boundaries. However, noncompliance with these no-effect boundaries was not considered evidence of a significant DDI. Statistical analyses were performed using SAS (version 9.4) and the mixed procedure. Adverse events were descriptively summarized.
