# Supplementary material for: Absence of Clinically Meaningful Drug-Drug Interactions with Rezafungin: Outcome of Investigations
Source: Microbiol Spectr. 2023 May 8;11(3):e01339-23. doi: 10.1128/spectrum.01339-23 (PMC10269561; doi:10.1128/spectrum.01339-23)
Supplement: Supplemental file 1 — Supplemental material. Download spectrum.01339-23-s0001.pdf, PDF file, 0.3 MB [file spectrum.01339-23-s0001.pdf]

Supplementary Materials:

Table S1 Demographic and baseline characteristics of Study 1 and Study 2 participants

Characteristic	Study 1		Study 2		
	Safety (N=26)	PK (N=26)	Cohort 1-Males (N=18)	Cohort 2-Females (N=16)	Total (N=34)
Age (years),					
Mean (SD)	39 (9.00)	39 (9.00)	37.9 (7.17)	39.3 (11.93)	38.6 (9.58)
Range	26–55	26–55	27–48	21–59	21–59
Gender, n (%)					
Male	24 (92.3)	24 (92.3)	18 (100)	0	18 (52.9)
Female	2 (7.7)	2 (7.7)	0	16 (100)	16 (47.1)
Race, n (%)					
White	11 (42.3)	11 (42.3)	6 (33.3)	9 (56.3)	15 (44.1)
Black or African American	15 (57.7)	15 (57.7)	12 (66.7)	7 (43.8)	19 (55.9)
Ethnicity, n (%)					
Hispanic or Latino	2 (7.7)	2 (7.7)	1 (5.6)	5(31.3)	6 (17.6)
Not Hispanic or Latino	24 (92.3)	24 (92.3)	17 (94.4)	11 (68.8)	28 (82.4)
Height (cm)					
Mean (SD)	177.00 (8.70)	177.00 (8.70)	178.81 (9.61)	161.97 (6.34)	170.88 (11.78)
Weight (kg)					
Mean (SD)	86.5 (11.00)	86.5 (11.00)	87.03 (13.51)	69.02 (8.87)	78.55 (14.60)
Body Mass Index (kg/m^2)					
Mean (SD)	27.5 (2.50)	27.5 (2.50)	27.18 (3.38)	26.30 (2.85)	26.76 (3.13)

Abbreviations: PK=pharmacokinetic; SD=standard deviation

Table S2 Summary of treatment-emergent adverse events for Study 1

System Organ Class<br>Preferred Term	Adverse Event Severity<br>Safety Population (N=26), n (%)			Rezafungin Safety Population (N=26), n (%)		
**Adverse Event**	**Mild**	**Moderate**	**Severe**	**Mild**	**Moderate**	**Severe**
**Number of Subjects with at least one TEAE**	12 (46.2%)	2 (7.7%)	0	11 (42.3%)	2 (7.7%)	0
**Blood and lymphatic system disorders**	1 (3.8%)	0	0	1 (3.8%)	0	0
Lymphadenopathy	1 (3.8%)	0	0	1 (3.8%)	0	0
**Gastrointestinal disorders**	8 (30.8%)	0	0	7 (26.9%)	0	0
Abdominal pain	3 (11.5%)	0	0	3 (11.5%)	0	0
Constipation	3 (11.5%)	0	0	0	0	0
Diarrhea	5 (19.2%)	0	0	5 (19.2%)	0	0
Feces hard	1 (3.8%)	0	0	0	0	0
Flatulence	1 (3.8%)	0	0	0	0	0
Nausea	1 (3.8%)	0	0	1 (3.8%)	0	0
**General disorders and administration site conditions**	5 (19.2%)	1 (3.8%)	0	5 (19.2%)	1 (3.8%)	0
Feeling hot	3 (11.5%)	0	0	3 (11.5%)	0	0
Infusion site extravasation	1 (3.8%)	0	0	1 (3.8%)	0	0
Infusion site pain	1 (3.8%)	1 (3.8%)	0	1 (3.8%)	1 (3.8%)	0
**Investigations**	1 (3.8%)	0	0	1 (3.8%)	0	0
Urine output increased	1 (3.8%)	0	0	1 (3.8%)	0	0
**Musculoskeletal and connective tissue disorders**	1 (3.8%)	0	0	1 (3.8%)	0	0
Arthralgia	1 (3.8%)	0	0	1 (3.8%)	0	0
**Nervous system disorders**	7 (26.9%)	0	0	7 (26.9%)	0	0
Dizziness	2 (7.7%)	0	0	2 (7.7%)	0	0
Headache	4 (15.4%)	0	0	3 (11.5%)	0	0
Somnolence	3 (11.5%)	0	0	3 (11.5%)	0	0
Tremor	1 (3.8%)	0	0	1 (3.8%)	0	0
**Psychiatric disorders**	1 (3.8%)	0	0	1 (3.8%)	0	0
Anxiety	1 (3.8%)	0	0	1 (3.8%)	0	0
**Respiratory, thoracic, and mediastinal disorders**	1 (3.8%)	0	0	1 (3.8%)	0	0
Nasal congestion	1 (3.8%)	0	0	1 (3.8%)	0	0
**Skin and subcutaneous tissue disorders**	2 (7.7%)	0	0	1 (3.8%)	0	0
Dermatitis contact	1 (3.8%)	0	0	0	0	0
Ecchymosis	1 (3.8%)	0	0	1 (3.8%)	0	0
**Vascular disorders**	1 (3.8%)	1 (3.8%)	0	1 (3.8%)	1 (3.8%)	0
Hot flush	1 (3.8%)	0	0	1 (3.8%)	0	0
Phlebitis	0	1 (3.8%)	0	0	1 (3.8%)	0

Abbreviations: TEAE = Treatment emergent adverse event. Safety Population = all subjects who receive any amount of study drug; Rezafungin Safety Population = all subjects who receive any amount of rezafungin for injection; each TEAE is counted only once for each subject within each System Organ Class and Preferred Term by using the adverse events with the highest intensity within each category.

Table S3 Summary of treatment-emergent adverse events reported by ≥2 Subjects Study 2

**System Organ Class, n (%)**<br>Preferred Term, n (%)	**Without rezafungin (N=34)**<br>n (%)	**With rezafungin (N=32)**<br>n (%)
**Nervous system disorders**	**3 (8.8)**	**6 (18.8)**
Headache	3 (8.8)	6 (18.8)
**Gastrointestinal disorders**	**5 (14.7)**	**3 (9.4)**
Nausea	3 (8.8)	1 (3.1)
Abdominal discomfort	0	2 (6.3)
Vomiting	2 (5.9)	0
**General disorders and administration site conditions**	**2 (5.9)**	**5 (15.6)**
Feeling hot	2 (5.9)	3 (9.4)
**Cardiac disorders**	**0**	**2 (6.3)**
Palpitations	0	2 (6.3)
**Vascular disorders**	**0**	**2 (6.3)**
Phlebitis	0	2 (6.3)

Abbreviations: TEAE = treatment-emergent adverse event.
N = number of subjects in the safety population; n = number of subjects in a sample.
Notes: Each TEAE was counted only once for each subject within each System Organ Class and Preferred Term.

Table S4 Summary of initial *in vitro* assessments

Study Number	Study Description	Study Results
NC-049	Membrane permeability and P-gp and/or BCRP substrate potential of rezafungin was assessed using Caco-2 epithelial monolayers expressing human P-gp and BCRP.	Permeability of rezafungin was very poor (permeability coefficient <0.2 x 10-6 cm/s) in both transport directions in the absence and presence of inhibitors. It was concluded that active transport was likely not to be involved and rezafungin was not a substrate of P-gp or BCRP.
NC-052	A study to determine if rezafungin is a substrate of human OATP1B1 and OATP1B3 transporters in transiently transfected HEK293 cells.	Rezafungin was determined not to be a substrate of OATP1B1 or OATP1B3 (uptake ratio <2).
NC-152	A study to assess the potential of rezafungin as an inhibitor of the human transporters P-gp, BCRP, OATP1B1, OATP1B3, OAT1, OAT3, OCT2, OCT1, MATE1 and MATE2-K in various *in vitro* cell test systems, and of BSEP in membrane vesicles.	Rezafungin was determined to be an inhibitor of probe substrate transport mediated via P-gp, OATP1B1, OATP1B3, OAT1, OCT2, OCT1, MATE1 and MATE2-K, but not via BCRP, OAT3 or BSEP. Potential cytotoxicity was observed at 10, 30, and 100 µM (Caco-2) or 30 and 100 µM (MDCK-MDR1) in polarized cell-based systems, and at 100 µM in SLC transporter cell systems.
NC-160	Evaluation of *in vitro* substrate interactions of rezafungin with BCRP, MDR1, and MRP2 Efflux (ABC) and OCT1, OCTN1, and OCTN2 uptake SLC transporter as well as the inhibition of rezafungin with BCRP using membrane vesicle preparations.	Rezafungin was not a substrate of BCRP, MDR1, MRP2, OCT1, OCTN1, or OCTN2. Further, in membrane vesicles, rezafungin inhibited BCRP with a maximum inhibition of 83% at 60 µM whereas inhibition was only 9% at 6 µM.
NC-012	CYP450 inhibition screening (at concentration of 10 µM) in incubations of fluorogenic substrates with recombinant human CYPs 1A2, 2B6, 2C8, 2C9, 2C19, 2D6 and 3A4	Minimal inhibition of CYP isoforms noted at 10 µM except for CYP2C8 (63%) and CYP3A4 (81%, [benzyloxyresorufin substrate]).
NC-013	Follow-up CYP450 inhibition study in human liver microsomes to further examine the potential for rezafungin to inhibit the activity of CYP isoforms 2C8 and 3A4.	In human liver microsomes, rezafungin weakly (IC_{50}>25 µM) inhibited activity of both CYP2C8 and 3A4.
NC-153	Study to determine whether rezafungin is a time dependent inhibitor of the CYP isoforms 1A2, 2B6, 2C8, 2C9, 2C19, 2D6 and 3A4 (midazolam and testosterone as substrates) in human liver microsomes.	Rezafungin was not a time-dependent inhibitor of CYP isoforms 1A2, 2B6, 2C8, 2C9, 2C19, 2D6 and 3A4 at concentrations ranging from 0.1 to 25 µM (precipitation occurred at ≥25 µM).
NC-161	CYP450 induction study using human hepatocytes to determine whether rezafungin can induce CYP isoforms 1A2, 2B6, or 3A4.	No evidence of CYP induction (as measured by <2-fold increase in mRNA expression) of CYP1A2 and CYP2B6, and, in the case of CYP3A4, in 2 of 3 donors. More specifically, for CYP3A4, only 1 out of the 3 donors tested showed a 2.68-fold induction of mRNA expression at the highest feasible concentration (3 µM) tested.

Abbreviations: ABC = ATP binding cassette; BCRP = breast cancer resistance protein; BSEP = bile salt export pump; CYP = cytochrome P450; MATE = multidrug and toxic compound extrusion; MDCK = Madin-Darby canine kidney; MDR = multidrug resistance protein; mRNA = messenger ribonucleic acid; OAT = organic anion transporter; OATP = organic anion transporter protein; OCT – organic cation transporter; OCTN = organic cation transporter novel; P-gp = P-glycoprotein; SLC = solute carrier.

Table S5 Schedule of pharmacokinetic assessments for Study 1

	**Time Points Relative to Administration of Drugs**																	
	**Minutes**				**Hours (±minutes)**													
**Drugs**	**≤60[a]**	**15 (±1)**	**30 (±5)**	**45 (±5)**	**1 (±10)**	**2 (±10)**	**3 (±10)**	**4 (±10)**	**6 (±10)**	**8 (±10)**	**12 (±30)**	**24 (±30)**	**48 (±30)**	**72 (±30)**	**96 (±30)**	**120 (±30)**	**144 (±30)**	**168 (±30)**
	**Tacrolimus, Repaglinide Without/With Rezafungin for Injection**																	
	**Day -21**											**Day -20**	**Day -19**	**Day -18**	**Day -17**		**Day -15**	
Tacrolimus 5 mg	X		X		X	X	X	X		X	X	X	X	X	X		X	
Repaglinide 1 mg	X	X	X	X	X	X	X	X	X	X	X	X						
	**Day 1**											**Day 2**	**Day 3**	**Day 4**	**Day 5**		**Day 7**	
Tacrolimus 5 mg	X		X		X[b]	X	X	X		X	X	X	X	X	X		X	
Repaglinide 1 mg	X	X	X	X	X[b]	X	X	X	X	X	X	X						
Rezafungin for injection 600 mg	X				X[c]	X	X	X		X		X		X	X		X[d]	
	**Metformin, Rosuvastatin Without/With Rezafungin for Injection**																	
	**Day -15**											**Day -14**	**Day -13**	**Day -12**	**Day -11**			
Metformin 500 mg	X		X		X	X	X	X	X	X	X	X	X					
Rosuvastatin 5 mg	X	X	X	X	X	X	X	X	X	X	X	X	X	X	X			
Pitavastatin 2 mg	X	X	X	X	X	X	X		X		X	X	X					
	**Day 10**											**Day 11**	**Day 12**	**Day 13**	**Day 14**			
Metformin 500 mg	X		X		X[b]	X	X	X	X	X	X	X	X					
Rosuvastatin 5 mg	X	X	X	X	X[b]	X	X	X	X	X	X	X	X	X	X			
Pitavastatin 2 mg	X	X	X	X	X[b]	X	X		X		X	X	X					
Rezafungin for injection 400 mg					X[c]										X[e]			
	**Caffeine, Efavirenz, Midazolam, Digoxin Without/With Rezafungin for Injection**																	
	**Day -9**											**Day -8**	**Day -7**	**Day -6**		**Day -4**		**Day -2**
Caffeine 100 mg	X	X	X	X	X	X	X	X	X	X	X	X						
Efavirenz 50 mg	X				X	X	X	X	X	X	X	X	X	X		X		X
Midazolam 2 mg	X	X	X	X	X	X	X	X	X	X	X							
Digoxin 0.25 mg	X		X		X	X	X	X		X	X	X	X	X		X		X
	**Day 15**											**Day 16**	**Day 17**	**Day 18**		**Day 20**		**Day 22**
Caffeine 100 mg	X	X	X	X	X[b]	X	X	X	X	X	X	X						
Efavirenz 50 mg	X				X[b]	X	X	X	X	X	X	X	X	X		X		X
Midazolam 2 mg	X	X	X	X	X[b]	X	X	X	X	X	X							
Digoxin 0.25 mg	X		X		X[b]	X	X	X		X	X	X	X	X		X		X
Rezafungin for injection 400 mg					X[c]													X[f]

[a]Within 60 minutes prior to the start of the infusion; [b]End of infusion draw for substrate drugs; [c]Within 10 minutes prior to the end of the infusion for rezafungin for injection; [d]At time of last pharmacokinetic blood draw for tacrolimus; [e]At time of last pharmacokinetic draw for rosuvastatin; [f]At time of last pharmacokinetic draws for efavirenz and digoxin

Table S6 Schedule of pharmacokinetic assessments for Study 2

**Cyclosporine without Rezafungin**																	
**Drugs**	**Time Points Relative to Administration of Drugs – hours (±minutes)**																
	**Pre-dose**	**0.5**	**1**	**1.5**	**2**	**2.5**	**3**	**4**	**6**	**8**	**9**	**12**	**24**	**48**	**72**	**96**	**168**
	**(-60)**	**(±5)**	**(±10)**									**(±30)**					
	**Day -16**												**Day -15**	**Day -14**	**Day -13**		
Cyclosporine	X	X	X	X	X	X	X	X	X		X	X	X	X	X		

**Cyclosporine with Rezafungin**																	
**Drugs**	**Time Points Relative to Administration of Drugs – hours (±minutes)**																
	**Pre-dose**	**0.5**	**1**	**1.5**	**2**	**2.5**	**3**	**4**	**6**	**8**	**9**	**12**	**24**	**48**	**72**	**96**	**168**
	**(-60)**	**(±5)**	**(±10)**									**(±30)**					
	**Day 1**												**Day 2**	**Day 3**	**Day 4**	**Day 5**	**Day 8**
Cyclosporine	X	X	X	X	X	X	X	X	X		X	X	X	X	X		
Rezafungin	X		X	X			X		X	X		X	X	X		X	X [a]

**Ibrutinib without Rezafungin**																	
**Drugs**	**Time Points Relative to Administration of Drugs – hours (±minutes)**																
	**Pre-dose**	**0.25**	**0.5**	**1**	**1.5**	**2**	**3**	**4**	**6**	**8**	**9**	**12**	**18**	**24**	**48**	**96**	**168**
	**(-60)**	**(±5)**		**(±10)**								**(±30)**					
	**Day -10**													**Day -9**	**Day -8**		
Ibrutinib	X	X	X	X	X	X	X	X	X		X	X	X	X	X		

**Ibrutinib with Rezafungin**																	
**Drugs**	**Time Points Relative to Administration of Drugs – hours (±minutes)**																
	**Pre-dose**	**0.25**	**0.5**	**1**	**1.5**	**2**	**3**	**4**	**6**	**8**	**9**	**12**	**18**	**24**	**48**	**96**	**168**
	**(-60)**	**(±5)**		**(±10)**								**(±30)**					
	**Day 8**													**Day 9**	**Day 10**	**Day 12**	**Day 15**
Ibrutinib	X	X	X	X	X	X	X	X	X		X	X	X	X	X		
Rezafungin	X [a]			X	X		X		X	X		X		X	X	X	X [a]

Mycophenolate Mofetil without Rezafungin

Drugs	Time Points Relative to Administration of Drugs – hours (±minutes)																				
	**Pre-dose**	**0.33**	**0.67**	**1**	**1.33**	**1.5**	**1.67**	**2**	**3**	**4**	**6**	**8**	**10**	**12**	**14**	**18**	**24**	**36**	**48**	**96**	**168**
	**(-60)**	**(±5)**		**(±10)**										**(±30)**							
	**Day -7**																**Day -6**		**Day -5**		
Mycophenolate mofetil	X	X	X	X	X		X	X		X	X	X	X	X	X	X	X	X	X		

Mycophenolate Mofetil with Rezafungin

Drugs	Time Points Relative to Administration of Drugs – hours (±minutes)																				
	**Pre-dose**	**0.33**	**0.67**	**1**	**1.33**	**1.5**	**1.67**	**2**	**3**	**4**	**6**	**8**	**10**	**12**	**14**	**18**	**24**	**36**	**48**	**96**	**168**
	**(-60)**	**(±5)**		**(±10)**										**(±30)**							
	**Day 15**																**Day 16**		**Day 17**	**Day 19**	**Day 22**
Mycophenolate mofetil	X	X	X	X	X		X	X		X	X	X	X	X	X	X	X	X	X		
Rezafungin	X [a]			X		X			X		X	X		X			X		X	X	X

Venetoclax without Rezafungin

Drugs	Time Points Relative to Administration of Drugs – hours (±minutes)																
	**Pre-dose**	**1**	**1.5**	**2**	**3**	**4**	**6**	**8**	**10**	**12**	**16**	**20**	**24**	**48**	**72**	**96**	**168**
	**(-60)**	**(±10)**									**(±30)**						
	**Day -7**												**Day -6**	**Day -5**	**Day -4**	**Day -3**	
Venetoclax	X	X		X		X	X	X	X	X	X	X	X	X	X	X	

Venetoclax with Rezafungin

Drugs	Time Points Relative to Administration of Drugs – hours (±minutes)																
	**Pre-dose**	**1**	**1.5**	**2**	**3**	**4**	**6**	**8**	**10**	**12**	**16**	**20**	**24**	**48**	**72**	**96**	**168**
	**(-60)**	**(±10)**									**(±30)**						
	**Day 15**												**Day 16**	**Day 17**	**Day 18**	**Day 19**	**Day 22**
Venetoclax	X	X		X		X	X	X	X	X	X	X	X	X	X	X	
Rezafungin	X [a]	X	X		X		X	X		X			X	X		X	X

Note: On each day of dosing, a low-fat standard meal was provided, to be completed 30 minutes prior to dosing. Only water was permitted until at least 4 hours post-dose.

[a]The 168-hour rezafungin samples on Days 8 and 15 were noted twice in the table (at 168 hours and at pre-dose for the next dosing period); they were the same sample, and were drawn prior to the start of the next dose of rezafungin for injection.
